# Functions and Clinical Significance of Super-Enhancers in Bone-Related Diseases

**DOI:** 10.3389/fcell.2020.00534

**Published:** 2020-06-30

**Authors:** Jian Qu, Zhanbo Ouyang, Wenqiang Wu, Guohua Li, Jiaojiao Wang, Qiong Lu, Zhihong Li

**Affiliations:** ^1^Department of Pharmacy, The Second Xiangya Hospital, Central South University, Changsha, China; ^2^Hunan Key Laboratory of Tumor Models and Individualized Medicine, The Second Xiangya Hospital, Central South University, Changsha, China; ^3^Mathematical Engineering Academy of Chinese Medicine, Guangzhou University of Chinese Medicine, Guangzhou, China; ^4^Department of Orthopedics, The Second Xiangya Hospital, Central South University, Changsha, China

**Keywords:** super-enhancers, bone-related diseases, osteosarcoma, Ewing sarcoma, multiple myeloma

## Abstract

Super-enhancers (SEs) are a large cluster of *cis*-regulatory DNA elements that contain many binding motifs, which master transcription factors and cofactors bind to with high density. SEs usually regulate the expression of genes that can control the cell identity and fate, and SEs can be used to explain the patterns of the expression of cell-specific genes. Hence, it shows great potential for application in the treatment of diseases like cancer. At present, the clinical treatments for osteosarcoma, Ewing sarcoma, and other bone-related diseases remain challenging. The poor prognosis and difficult treatment of these diseases imposes heavy economic burden on patients and society. In recent years, research on SEs with respect to bone-related diseases has attracted increasing attention. In this paper, we first review the identification and functional mechanisms of SEs. Then, we integrate the findings of the emerging studies on SEs in bone-related diseases. Finally, we summarize recent strategies for targeting SEs for the treatment of bone-related diseases. This review aims to provide comprehensive insights into the roles of SEs in bone-related diseases.

## Introduction

**FIGURE 1:**
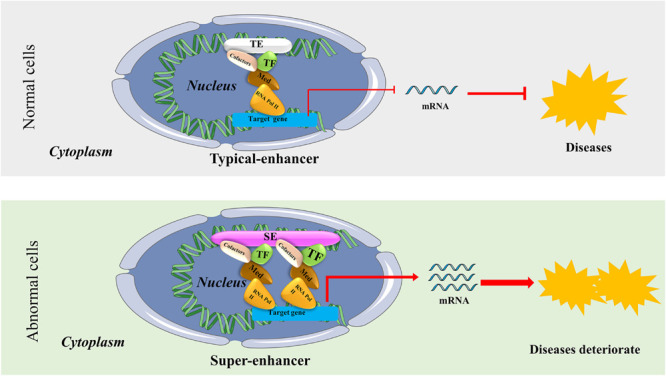
The structure and possible functional mechanism of super-enhancers (SEs) vs. typical enhancers (TEs). SEs are a large cluster of *cis*-regulatory DNA elements densely bound by transcription factors and cofactors. SEs are often found near genes that have cell type-specific functions and genes closely associated with the progress of bone-related diseases. It usually causes diseases or accelerates the progress of diseases by regulating the expression of disease-specific genes.

Since the Human Genome Project was launched in 1990, our understanding of the regulatory mechanism of genes has evolved drastically. Nowadays, many scientists believe that gene transcription is controlled by types of regulatory elements known as enhancers and super-enhancers (SEs). Bulger and Groudine, 2011 proposed that enhancers are functionally defined as non-coding elements that can activate gene transcription through long-range *cis* chromatin interactions. Initially, enhancers only meant the typical enhancers (TEs). However, Hnisz et al. (2013) proposed the concept of SEs in 2013. They put forward that although TEs and SEs are similar with respect to carrying the same components such as transcription factors (TFs), cofactors, mediator complex, and RNA polymerase II (pol II) complex, SEs harbors these factors on an average 10-fold higher density than do TEs (Hnisz et al., 2013; Whyte et al., 2013; Thandapani, 2019). The difference between the function and structure of TEs and SEs are shown in Figure 1. SEs are a large cluster of *cis*-regulatory DNA elements densely bound by transcription factors and cofactors and play critical roles in defining cell fate and identity (Hnisz et al., 2013). Histone marks such as H3K27ac, H3K4me1, and the transcription cofactor p300 are often used to define SEs (Riggi et al., 2014; Kennedy et al., 2015; Niederriter et al., 2015). Ever since their discovery, SEs have been the focus of increasing research to investigate their roles in cancers and inflammatory diseases like osteoarthritis. For example, previous research indicated that SEs frequently drive high-level expression of prominent oncogenes in cancer cells (Hnisz et al., 2013; Chipumuro et al., 2014; Kwiatkowski et al., 2014). Other groups found that SEs play a critical role in autoimmune diseases like juvenile idiopathic arthritis (Peeters et al., 2015). Shin (2018) proposed that SEs can be used as biomarkers to develop novel disease diagnostic tools and establish new directions in clinical therapeutic strategies. Hence, SEs can be considered novel targets in diseases, and targeting SEs should be a promising therapeutic strategy.

The clinical treatment of bone-related diseases such as osteosarcoma, Ewing sarcoma, osteoporosis, cartilage dysplasia, chordoma, rheumatoid arthritis, osteoarthritis, and multiple myeloma continue to remain challenging. Although the incidence of these bone-related diseases is relatively lower than cancers like lung cancer, the poor prognosis and difficult treatment imposes a heavy burden on patients and caregivers. To address this complex problem, researchers have aimed to develop high-efficiency, novel therapies based on targeting SEs to treat bone-related diseases. In recent years, the functional mechanism of SEs in these bone-related diseases has attracted increasing attention. Thus, this review will mainly focus on integrating the findings of the emerging studies on SEs (shown in Table 1) in bone-related diseases and summarizing the strategies of targeting SEs as a clinical treatment for bone-related diseases. We believe it can provide new insights and angles for future studies and optimum treatment strategies using SEs in bone-related diseases.

**TABLE 1:** Summary of the researches about the SEs in bone-related diseases.

Diseases*	Cell/tissue*	SEs-targeted genes	Methods	Findings	References
OS	OS cell lines (U2OS/SJSA-1)	*MYC, STAT3*	ChIP-Seq of H3K27ac	THZ2 can inhibit the progression of osteosarcoma by targeting SE-associated genes like *MYC*, *STAT3*	Zhang et al., 2020
OS	OS cell lines (143B and SJSA1), primary OS cells (ZOS and ZOSM), and OS tissue	*LIF*	ChIP-Seq of H3K27ac	SEs function as an activator of NOTCH1 pathway through regulating LIF/STAT3 pathway in promoting the progression of osteosarcoma	Lu et al., 2020
ES	ES cell lines (A673/SKNMC)	*BCL11B, GLG1*	Analysis of ChIP-Seq data of H3K27ac	SEs-targeted genes *BCL11B* and *GLG1* are novel biomarkers for the diagnosis of Ewing sarcoma	Baldauf et al., 2018
ES	ES cell lines (A673/SKNMC)	*MEIS1*	ChIP-Seq of H3K27ac	SEs-associated gene *MEIS1* promotes transcriptional dysregulation in Ewing sarcoma in co-operation with EWS-FLI1	Lin et al., 2018
ES	ES cell lines (TC32/TC71)	*CCND1*	ChIP-Seq of H3K27ac	SEs-associated gene *CCND1* promotes the progression of Ewing sarcoma	Kennedy et al., 2015
ES	ES cell lines-A673 cells treated with JQ1	*EWS-FLI1*	ChIP of BRD4 and analysis of ChIP-Seq data of H3K27ac	SE-inhibitors like JQ1 can repress the progression of Ewing sarcoma through impacting *EWS-FLI1*’ expression	Jacques et al., 2016
Chordoma	Chordoma cell lines and chordoma tissue	*TBXT*	ChIP-Seq of H3K27ac	The dysregulation of *TBXT* regulated by SEs is the main cause for the tumorigenesis of chordoma	Sharifnia et al., 2019
MM	Primary cells isolated from MM tissue and Myeloma cell lines	*IRF4, FLI1*	ChIP-Seq of H3K27ac	*IRF4* and *FLI1* showed significant overlap with SEs. They can promote the progress of MM	Jin et al., 2018
MM	MM1.S MM cells	*MYC*	ChIP-Seq of H3K27ac, BRD4, MED1	SE-inhibitors treatment like JQ1 results in preferential loss of BRD4 in *MYC*-SE, which can repress the progress of MM	Loven et al., 2013
OP	GM12878 cells/osteoblast	*RANKL*	Analysis of ChIP-Seq data of H3K27ac from ENCODE	The dysregulated expression of the SE-associated gene *RANKL* is critical for the progress of osteoporosis	Zhu et al., 2018
CD	Rat chondrosarcoma cells	*SOX9, SOX6, SOX5*	ChIP-Seq 0f H3K27ac	The dysregulation of the SE- associated genes may cause cartilage dysplasia	Liu and Lefebvre, 2015
RA	CD4 + T cell	*BACH2*	ChIP-Seq of P300 protein	SEs-associated gene *BACH2* which is critical for the progress of RA	Vahedi et al., 2015
OS	Chondrocyte cell (sw1353 and chondrocytes isolated from OA tissue)	*HMGB1*	ChIP of BRD4 and analysis of ChIP-Seq data of H3K27ac	SEs may mediate the BRD4 regulating role in the expression of *HMGB1*	Jiang et al., 2017

*OS cell, osteosarcoma cell; ES cell, Ewing sarcoma cell; OP cell, osteoporosis cell; CD cell, cartilage dysplasia cell; chordoma, RA cell, rheumatoid arthritis cell; OA cell, osteoarthritis cell; and MM cell, multiple myeloma cell.

## The Identification of SEs

Diseases-related cells acquire SEs through various mechanisms, including (i)mutations and genomic alterations like deletions, duplications, translocations, insertions, inversions (Mansour et al., 2014; Oldridge et al., 2015; Zhang et al., 2019); (ii) single-nucleotide polymorphisms (SNPs) (Zhu et al., 2018); (iii) chromosomal rearrangements (Affer et al., 2014; Gröschel et al., 2014; Demchenko et al., 2016); and (iv) 3D genome structural changes (Furlong and Levine, 2018). Once the SEs originated through the above mechanisms, resulting in the dysregulation of nearby SEs-target genes, which ultimately accelerates the deterioration of diseases.

With rapid developments in genetic research, many refractory diseases with complex disease mechanisms have been well studied. However, despite researchers having identified some effective treatments, osteosarcoma, Ewing sarcoma, and other bone-related diseases remain difficult to treat. The poor prognosis and difficulty in treatment impose heavy economic burden on patients and society. Although many studies have proposed different treatment options for bone-related diseases, the effects of these treatments are not satisfactory. Therefore, significant research is on-going in an attempt to discover satisfactory treatment methods for these diseases.

Loven et al. (2013) and Whyte et al. (2013) first identified SEs in mouse embryonic stem cells and human cells. Since then, several publications have cited the SEs and identified its applications in different diseases (Chapuy et al., 2013; Loven et al., 2013; Whyte et al., 2013; Brown et al., 2014; Vaharautio and Taipale, 2014). Besides, an SE database—dbSUPER—in the mouse and human genome has been established by Khan and Zhang (2016). It is the first integrated and interactive database of SEs and serves as a reference for further studies related to transcriptional control of cell identity and disease. Pott and Lieb (2015) summarized the characteristics of SEs based on these publications: (i) SEs are enriched for motifs related to cell type-specific master transcription factors, and are often found near genes that have cell type-specific functions, including known master regulators; (ii) SEs are enriched for trait-associated variants; (iii) SEs overlap with previously defined large-scale regulatory domains and (iv) SEs in cancer cells are enriched in the vicinity of oncogenes. Summarizing from previous studies, abnormal SEs in disease states may form because of focal amplification, genomic regulatory elements rearrangements (Affer et al., 2014; Walker et al., 2014), and small insertions and deletions.

In recent years, research on SEs in diseases has become an important focus of clinical and basic research. As for the functional mechanism of the SEs (shown in Figure 1), Sengupta and George (2017) concluded that transcription factor (TF) binding to enhancers results in recruitment of the mediator complex. This, in turn, facilitates enhancer interaction with the basal transcription machinery and RNA polymerase II (Pol II) at the promoter regions in a gene-specific manner, a process mediated by “looping” of the loaded enhancer to the cognate promoter (Hnisz et al., 2016; Sur and Taipale, 2016). Thus, SEs can influence disease progression by initiating downstream transcription of the affected genes (Sengupta and George, 2017).

## SEs in Bone-Related Diseases

### SEs in Osteosarcoma

Osteosarcoma (OS) is the most common primary sarcoma occurring in children and adolescents. Metastasis and recurrence of osteosarcoma are the main influencing factors for the poor survival rate. The overall 5-year survival rate for patients with non-metastatic osteosarcoma is approximately 60–70%, while that for patients with metastatic and recurrent osteosarcoma is significantly reduced (Kempf-Bielack et al., 2005; Luetke et al., 2014; Kwiatkowski et al., 2014). Researchers have found that SEs play a critical role in the progression of osteosarcoma by promoting its growth and metastasis.

Chen et al. (2018) found a significant increase in *MYC* expression in metastatic osteosarcoma samples, suggesting that *MYC* may be an important therapeutic target for osteosarcoma treatment. They further tested the recently developed small-molecule SE-inhibitors of osteosarcoma and found that the SE-inhibitors were effective in inhibiting the proliferation, migration, and invasion of osteosarcoma cells. This is likely due to the inhibition of a large population of SEs containing the *MYC* target gene by THZ1 [a SE-inhibitor that also functions as a CDK7 (cyclin-dependent kinase) inhibitor] treatment. They also reported that the chosen sensitivity of some SE genes are more sensitive to THZ1 treatment than others, indicating that *MYC* binding strength, SE size and intensity, basic gene expression levels, and the activity of transcription factors and cofactors may lead to differential responses of the SEs gene to THZ1 treatment. Chen et al. (2018) have experimentally demonstrated that SE-inhibitors are promising small-molecule drugs for patients with osteosarcoma by targeting the MYC signaling pathway in osteosarcoma.

Zhang et al. (2020) found THZ2 can target SE-associated oncogenes in osteosarcoma. Using ChIP-Seq, they found that SEs are associated with oncogenic genes in osteosarcoma cells. They further reported several SE-associated genes to be sensitive to THZ2 treatment. Finally, they proved their hypothesis that THZ2 can inhibit the progression of osteosarcoma by targeting SE-associated genes such as *MYC*, *STAT3*, and *HMOX1*. These findings indicate that THZ2 has the potential to become a promising targeting drug for osteosarcoma treatment.

Morrow et al. (2018) suggested that enhancer elements can confer metastatic ability to osteosarcoma cells. They found that metastatic osteosarcoma and non-metastatic osteosarcoma have different enhancer activities for metastatic genes, and proposed a new concept of Metastatic Variant Enhancer Loci (Met-VELs). Comparing the activity of the gene with Met-VELs with the activity of SE-associated genes, they proved the existence of different Met-VELs in related metastasis genes, which lead to differences in metastatic ability between metastatic and non-metastatic osteosarcoma. Further, genes with Met-VELs were reportedly more related to osteosarcoma metastasis, while those with SE-associated genes were more related to the tumorigenesis and survival of osteosarcoma.

Lee et al. (2015) found the following phenomenon in the study of the treatment mechanism of JQ1—a bromodomain and extra terminal domain (BET) protein inhibitor—in human osteosarcoma: although the inhibition of bromodomain-containing protein 4 (BRD4) by JQ1 could inhibit the growth of osteosarcoma cells *in vitro*. They showed that the level of c-MYC protein in the cells treated with JQ1 remained unchanged. Meanwhile, some studies showed that JQ1 treatment significantly decreased the expression of *RUNX2* gene, which can be regulated by SEs with the region enriched for H3K27Ac (Encode Project Consortium, 2004; Loven et al., 2013; Whyte et al., 2013). Thus, Lee et al. (2015) suggested that the downstream target gene of JQ1 in osteosarcoma is *RUNX2* but not *MYC*, and they proved it through experiments such as ChIP-seq assay.

**FIGURE 2:**
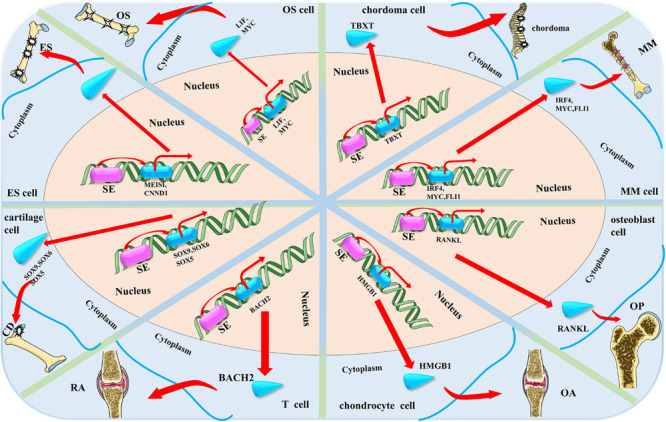
The possible mechanism of super enhancers (SEs) in the eight main bone-related diseases discussed in this review. SE accelerates the progress of the four bone-related diseases by activating or up-regulating the expression of bone-related disease-specific genes that are near the related SEs (OS, osteosarcoma; ES, Ewing sarcoma; OP, osteoporosis; CD, cartilage dysplasia; RA, rheumatoid arthritis; OA, osteoarthritis; and MM, multiple myeloma).

By profiling the epigenetic characteristics and SEs landscape of the osteosarcoma and some clinical specimens, Lu et al. (2020) found the SE-mediated regulator LIF can promote osteosarcoma stemness gene expression. Mechanistically, they found SEs function as an activator of the NOTCH1 pathway through regulating the LIF/STAT3 pathway in promoting the progression of osteosarcoma. They believe that their findings will provide new insights for the treatments of osteosarcoma. The possible functional mechanism of SEs in osteosarcoma is shown in Figure 2.

### SEs in Ewing Sarcoma

Ewing sarcoma is the second most common and devastating primary malignant tumor, and mainly occurs in children and adolescents. Despite advances in treatment over the past few decades, patient survival remains unsatisfactory. Thus, there is an urgent need for more effective treatments based on the molecular basis of cancer (Riggi and Stamenkovic, 2007; Mackintosh et al., 2010; Stoll et al., 2013). Several studies have shown that SEs play a critical role in the tumorigenesis, proliferation, and metastasis of Ewing sarcoma (Kennedy et al., 2015; Jacques et al., 2016; Baldauf et al., 2018; Lin et al., 2018).

Recent studies have indicated that the disease-associated fusion protein EWS-FLI1 activates or inhibits enhancer elements in Ewing sarcoma through different chromatin remodeling patterns (Riggi et al., 2014; Tomazou et al., 2015). Baldauf et al. (2018) found out that the EWSR1-ETS-targeted genes—*BCL11B* and *GLG1*—are driven by SEs through immunohistochemistry analysis. They found that both *BCL11B* and *GLG1* are more specific than *CD99* for Ewing sarcoma, suggesting that *BCL11B* and *GLG1* can be used as novel biomarkers for the diagnosis of Ewing sarcoma. Therefore, the discovery of *BCL11B* and *GLG1* may significantly reduce the number of misdiagnosed patients, thereby improving the care of patients with Ewing sarcoma.

Lin et al. (2018) used epigenetic genomics analysis to characterize transcriptional disorders in Ewing sarcoma. By focusing on SEs and its associated transcriptional regulatory mechanisms, they demonstrated that SE-associated transcripts are significantly enriched in the *EWS-FLI1* target gene, which contributes to aberrant transcription in Ewing sarcoma and mediates the specific sensitivity of Ewing sarcoma to transcriptional repression. Through comprehensive analysis, the authors identified *MEIS1* as a SE-driven oncogene that synergizes with *EWS-FLI1* in transcriptional regulation and plays a key role in pro-survival in Ewing sarcoma. Besides, they identified that the SE-related gene *APCCD1*, a downstream target of *MEIS1* and *EWS-FLI1*, was also a novel tumor-promoting factor for Ewing sarcoma.

The main known carcinogenic event in Ewing sarcoma is somatic chromosomal translocation that occurs most commonly between chromosomes 11 and 22 and results in a fusion between the 5′ region of the *EWS* gene and the 3′ region of the ETS family gene *FLI1* (Delattre et al., 1992). Kennedy et al. (2015) suggested that while direct targeting of aberrant transcription factors remains a pharmacological challenge, identifying the dependence of *EWS/FLI1* expression will provide new therapeutic avenues. They used SE analysis, near-genome shRNA assay, and small-molecule screening to identify cyclin D1 and CDK to characterize the selective dependence of Ewing sarcoma. They found that SEs regulate cyclin D1 and promote its expression in Ewing sarcoma. Moreover, Ewing sarcoma cells require CDK4 and cyclin D1 to survive and do not rely on adherent growth. Pharmacological inhibition through CDK4 with selective CDK4/6 inhibitors results in cytostasis and cell death of Ewing sarcoma cell lines *in vitro* as well as growth retardation in the Ewing sarcoma xenograft model *in vivo*. These results showed the dependence of Ewing sarcoma on CDK4 and cyclin D1; hence, CDK4/6 inhibitors can be explored further for the treatment of patients with Ewing sarcoma.

Jacques et al. (2016) focused on the *EWS-FLI1* gene, the most popular target of Ewing sarcoma, and inspired by related research, hypothesized that the expression of *EWS-FLI1* gene may be regulated by the SEs and activated by BET bromodomain activity. Based on this, they proposed that JQ1 can directly target the *EWS-FLI1* gene, releasing BRD4 from the *EWS-FLI1* promoter, thereby resulting in down-regulation of *EWS-FLI1* gene expression and inhibiting the progression and metastasis of Ewing sarcoma (Jacques et al., 2016). They proved that JQ1 can down-regulate the expression of *EWS-FLI1*, as well as inhibit the downstream gene expression of *EWS-FLI1* through a series of cell proliferation, qRT-PCR, western blotting, and CHIP analysis experiments. Hence, it was proposed as a good therapeutic drug for Ewing sarcoma. The possible functional mechanism of SEs in Ewing sarcoma is shown in Figure 2.

### SEs in Chordoma

Chordoma is a major bone tumor that originates from the residual spinal cord tissue and occurs in the skull base, spine, and tibia (Salisbury, 1993; McMaster et al., 2001). Chordoma usually presents as a slow-growing but locally aggressive malignant tumor. There is still a recurrence tendency after surgery and radiotherapy. Given the inadequate understanding of the disease mechanism of chordoma, patients with advanced disease usually show poor prognosis because of the lack of effective therapies (Barry et al., 2011; Stacchiotti and Sommer, 2015).

Sharifnia et al. (2019) identified a SE-associated oncogenic TF named TBXT (developmental transcription factor T) with high expression in chordoma using comprehensive methods such as CRISPR-Cas9 screening, RNA-seq, and RT-qPCR. They found that CDK inhibitors can inhibit the proliferation of chordoma cells. They further demonstrated that TBXT is a SE-associated TF in chordoma. Similar to other diseases, SEs are associated with genes (*TBXT* in chordoma) that are critical for disease identity and maintenance. The dysregulation of *TBXT* regulated by SEs is the main feature of the regulatory environment in chordoma cells (Sharifnia et al., 2019). The possible functional mechanism of SEs in chordoma is shown in Figure 2.

### SEs in Multiple Myeloma

Multiple myeloma (MM) is a malignant tumor that originates from antibody-secreting plasma cells and is driven by many genetic and epigenetic changes (Landgren et al., 2009; Weiss et al., 2009; Kuehl and Bergsagel, 2012). Research efforts have revealed the involvement of SEs-associated genes such as *IRF4* (Jin et al., 2018) and *MYC* (Loven et al., 2013; Affer et al., 2014). Studies showed that these SEs-associated genes are critical for the tumorigenesis and development of MM.

Amorim et al. (2016) proposed that bromodomain binds to the acetylated lysine residue on the histone tail, allowing the extension complex that binds to the terminal portion of the protein to enter the promoter region of the gene, triggering gene transcription. Fulciniti et al. (2018) discovered that non-overlapping control of the promoter and SE-dependent transcriptomes in MM contributed to the growth and proliferation of MM. They also discovered the relationship between the transcription factor E2F and the transcriptional coactivator BRD4 in MM. They demonstrated the existence of two different regulatory pathways and showed that they can synergistically promote the growth and proliferation of MM. The discovered transcriptional regulatory mechanisms provide a theoretical basis for the development of new treatments for MM and other malignancies.

Affer et al. (2014) proposed that the *MYC* locus in MM has a loose rearrangement of enhancers and SEs that causes dysregulation of *MYC*. They proposed that rearrangement like this reposition of *MYC* nearby the genes related to SEs accelerates the progress of MM (Affer et al., 2014).

Although MM is well characterized in terms of genome and transcription, the abnormal regulatory network of the gene that supports this disease is still not well understood. Jin et al. (2018) described the regulatory elements and TF footprint in primary MM cells by RNA-seq and ChIP-seq assay. They established a SE-related TF-based regulatory network and identified several novel TFs critical for the biology of MM based on the newly established regulatory network. Their study indicated that *IRF4* and *FLI1* showed significant overlap with 92% of all SEs in MM cells and could promote the growth and proliferation of MM. These findings will provide potential therapeutic targets and new solutions for the treatment of MM.

Chromatin regulatory factors have become attractive targets for cancer therapy, but the mechanisms involved in inhibiting the gene-specific effects of tumor cells caused by these common regulatory factors are not well understood yet. Loven et al. (2013) investigated the mechanisms involved in the selective inhibition of *MYC* oncogenes in MM caused by inhibition of transcriptional coactivator BRD4. They found that treatment of MM tumor cells with the BET domain inhibitor, JQ1, resulted in preferential loss of BRD4 in *MYC*-SEs and subsequent transcriptional extension defects, which preferentially affected SE-associated genes such as *MYC*. This also consequently inhibited the progression of MM. The possible functional mechanism of SEs in MM is shown in Figure 2.

### SEs in Osteoporosis

Osteoporosis is a metabolic disease caused by reduced bone formation and/or increased bone resorption, which results in decreased bone mass and increased bone fragility. It is characterized by reduced bone mineral density (BMD) and low bone mass. Osteoporosis has become a global health concern. Zhu et al. (2018) found a SE-associated gene—*RANKL*—was critical for the progress of osteoporosis, by analyzing the ChIP-seq Data from ENCODE.

Some significant genome-wide association studies (GWAS) have shown that single nucleotide polymorphisms (SNPs) near *RANKL* are associated with osteoporosis on chromosome 13q14.11 (Zhang et al., 2014; Kuosmanen et al., 2016). Zhu et al. (2018) identified five BMD-associated SNPs (rs9533094, rs9533090, rs9594738, rs8001611, and rs9533095) that may be related to osteoporosis in the SEs region via bioinformatics data analysis and experimental validation. *RANKL* is a key regulator involved in bone remodeling and is critical for osteoclast differentiation, activation, survival, and enhancement of bone resorption (Lacey et al., 1998; Hsu et al., 1999; Jimi et al., 1999; Sobacchi et al., 2007). They proposed that these five SNPs up-regulate the expression of *RANKL* through long-range chromosomal interaction with SEs, thereby affecting bone metabolism and resulting in an imbalance between bone resorption and bone formation, leading to osteoporosis. These findings are critical to understanding the pathogenesis of osteoporosis. Research about *RANKL* will provide a new approach to the treatment of osteoporosis. The possible functional mechanism of SEs in osteoporosis are shown in Figure 2.

### SEs in Cartilage Dysplasia

During the development of vertebrate embryo, chondrocytes build hundreds of vital cartilage anlagen that provide morphological and skeletal support. Their unique elongation property can rapidly drive body growth (Liu and Lefebvre, 2015). Cartilage dysplasia (CD) can seriously affect people’s bone formation and growth (Warman et al., 2011). If cartilage dysplasia occurs in childhood, it will lead to lifelong defects like dwarfism or may even be fatal. Therefore, it is necessary to clarify the mechanisms related to cartilage development. Ohba et al. (2015) reported that *SOX9*-related SEs up-regulate the expression of *SOX9* gene which can promote cartilage formation. Liu and Lefebvre (2015) showed that three SOX proteins (SOX9, SOX6, and SOX5) play critical roles in chondrocyte development through SEs.

Using ChIP-seq, Ohba et al. (2015) discovered that the upstream region of key cartilage-related genes such as *SOX9* binds to a large number of enhancers and is rich in H3K27ac protein (characteristic of SEs). They found that the expression of the SEs-related genes (key cartilage-associated genes such as *SOX9*) is significantly higher than those associated with TEs. With reference to the above findings, Symon and Harley (2017) speculated that *SOX9*-related SEs up-regulate the expression of *SOX9* gene that can promote cartilage formation.

Liu and Lefebvre (2015) studied the target genes and functional modes of three SOX proteins (SOX9, SOX6, and SOX5) on chondrocytes using GWAS. They used rat chondrosarcoma (RCS) cell lines as a reliable model for chondrocytes and found that SOX6 and SOX9 bind thousands of genomic loci that are often close to each other. Although SOX proteins bind to a small number of TEs, they bind multiple sites on almost all SEs in RCS cells. These SEs are primarily associated with cartilage-specific genes. The three SOX proteins work together to activate these SEs. It is required for *in vivo* expression of their associated genes. These genes encode key regulatory factors including these three SOX proteins, as well as all essential cartilage extracellular matrix components. Chst11, Fgfr3, Runx2, and Runx3 are new targets for the three identified SOX proteins. Therefore, they proposed that these three SOX proteins closely cooperate with each other in the whole genome and effectively activate a large number of chondrocyte-specific genes through SEs, thereby realizing the unique differentiation and regulation process of chondrocytes. The possible functional mechanism of SEs in cartilage dysplasia are shown in Figure 2.

### SEs in Rheumatoid Arthritis

Rheumatoid arthritis (RA) is a chronic inflammatory synovitis systemic disease of unknown etiology. It is characterized by multiple, symmetric, and invasive arthritis of the joints of the hands and feet, often accompanied by extra-articular involvement and serum positivity for rheumatoid factor, which can lead to joint deformity and loss of function. Vahedi et al. (2015) found a SEs-associated gene *BACH2* critical for the progress of RA.

Vahedi et al. (2015) mapped the T-cell SEs through a series of experiments. They found that many cytokines and cytokine receptor genes with SEs structure are essential for the process of RA. *BACH2*, the gene encoding the key negative regulator of effector differentiation, is the most prominent T-cell SE. This reveals a network by which SE-related genes essential for T cell biology are inhibited by *BACH2*. Immune-mediated diseases (including RA) typically occur when disease-related SNPs are highly enriched in T-cell SEs rather than TEs. Treatment of T cells with the JAK inhibitor tofacitinib disproportionately alters the expression of RA risk genes with SEs structure. Based on these findings, the depiction of SEs can accurately identify key regulatory nodes in T cells that can be preferentially regulated by pharmacological interventions. It may provide new targets for the treatment of RA or other diseases. The possible functional mechanism of SEs in RA are shown in Figure 2.

### SEs in Osteoarthritis

Osteoarthritis is a common age-related degenerative disease that causes severe joint pain and physical dysfunction and is one of the important causes of disability in the elderly population (Xing et al., 2016). Despite its high incidence, the disease mechanism is poorly understood. Therefore, clarifying its etiology and pathogenesis can provide new drug targets for the clinical treatment of the disease. Jiang et al. (2017) found that SE inhibitor JQ1 can inhibit the progression of osteoarthritis by regulating the expression of *HMGB1*.

A growing number of studies have reported an important role for BRD4 in the pathology of inflammatory diseases which suggest its enormous potential as a drug target. Jiang et al. (2017) found that the BRD4 inhibitor, JQ1, inhibits the progression of osteoarthritis through dual effects on HMGB1 and NF-κB signaling pathways, proving that BRD4 may be a new target for the treatment of osteoarthritis. They found that BRD4 is enriched in the upstream non-promoter region of *HMGB1* by using the ChIP assay. In addition, treatment with JQ1 induced BRD4 release from the non-promoter and binding sites of *HMGB1*. They also found abundant enrichment of H3K27ac in the *HMGB1* promoter and upstream non-promoter region. Therefore, they speculated that the SEs may mediate the regulatory role of BRD4 in the expression of HMGB1. However, they could only prove that BRD4 may be a new target for the treatment of osteoarthritis, as the mechanism of SEs on BRD4 and HMGB1 was not investigated in their study. The possible functional mechanism of SEs in osteoarthritis are shown in Figure 2.

## Targeting SEs for Bone-Related Disease Therapy

Notably, as SEs harbor TFs binding sites with high density, a small change in the number of binding TFs can cause significant changes in associated gene transcription. Thus, disruption of the SEs structure and function should be the focus of disease therapy. Besides, as bone-related diseases prefer hijacking SEs to drive their aberrant transcription program which promotes disease processes like growth and metastasis, SEs have been exploited as a drug target with great potential for clinical treatment of bone-related diseases. Therefore, many researchers proposed that SEs can be considered as therapeutic targets. Accordingly, several types of small molecule inhibitors have been developed for the clinical treatment of bone-related diseases (shown in Table 2). Thus far, BET inhibitors and CDK inhibitors are the most widely used small molecule inhibitors (Jia et al., 2019). To a certain extent, drugs that target key components of SEs, such as BRD4 and CDK7, provide a novel strategy for better treatment of bone-related diseases.

**TABLE 2:** Inhibitor which targeting SEs in bone-related diseases.

Diseases	Inhibitor	Target of the inhibitor	Effect on SEs-driven transcription	Effects of SEs inhibition on diseases	References
Multiple myeloma	JQ1	BRD4	Decrease of BRD4 binding at SEs and downregulation of SEs associated genes	Repression of the progression of multiple myeloma	Loven et al., 2013
Osteosarcoma	JQ1	BRD4	Restraining of the expression of *MYC*	Inhibition of the growth and proliferation of osteosarcoma	Chen et al., 2018
Osteosarcoma	JQ1	BRD4	Restraining of the expression of *RUNX2*	Inhibition of the growth and proliferation of osteosarcoma	Lee et al., 2015
Osteosarcoma	THZ1	CDK7	Downregulation of SEs associated genes	Suppressing the proliferation, migration of osteosarcoma	Chen et al., 2018
Chordoma	THZ1	CDK7	Downregulation of SEs associated genes *TBXT*	Suppressing the proliferation of chordoma	Sharifnia et al., 2019
Osteosarcoma	THZ2	CDK7	Downregulation of SEs associated genes *LIF*, *STAT3*, *NOTCH1*	Impeding the proliferation and metastasis of osteosarcoma	Zhang et al., 2020
Ewing sarcoma	LEE011	CDK4/6	Downregulation of SEs associated genes	Impairing the progression of Ewing sarcoma	Kennedy et al., 2015
Osteoarthritis	JQ1	BRD4	Downregulation of SEs associated gene *HMGB1*	Inhibiting the progression of osteoarthritis	Jiang et al., 2017

### BET Inhibitors Targeting SEs

The BET family proteins including BRD2, BRD3, BRD4, and BRDT play a key role in chromatin remodeling and transcriptional regulation. JQ1, the first BET inhibitor applied to target BET bromodomain was discovered by Filippakopoulos et al. (2010). Recently, many studies have shown that JQ1 has anti-proliferative activity against many cancers, and it exhibits anti-proliferative activity mainly via inhibiting c-MYC expression and up-regulating p21 expression (Delmore et al., 2011; Dang, 2012). Jia et al. (2019) concluded that JQ1 treatment displaces BRD4 preferably from histone proteins and disrupts SEs. Partly, the mechanism by which BET inhibitors like JQ1 inhibit bone-related disease progression is through repressing the SE-associated genes that are critical for bone-related diseases. As mentioned above, JQ1 treatment on MM cells decreased BRD4 binding at SEs and subsequently repressed the SE-associated genes of MM (Loven et al., 2013). It is also reported that JQ1 can inhibit the growth and disease process of osteosarcoma by repressing the SEs containing oncogenes by limiting *MYC* expression (Chen et al., 2018). However, others found that JQ1 treatment inhibits the growth and disease process of osteosarcoma by targeting *RUNX2* instead of *MYC* through influencing SEs (Lee et al., 2015). Another study initiated by Jacques et al. proposed that the use of JQ1 can repress the expression of *EWS-FLI1* by disrupting the *EWS-FLI1*-associated SEs and subsequently control the disease process in Ewing sarcoma (Jacques et al., 2016). Besides, Jiang et al. (2017) showed that JQ1 treatment-induced BRD4 release from the non-promoter and binding sites of *HMGB1*. They also hypothesized that it can inhibit the progression of osteoarthritis. In conclusion, SEs are sensitive to bromodomain inhibition. BET inhibitors have great therapeutic potential in bone-related diseases.

Up to now, researchers have initialed clinical trials about BET inhibitors applied in treatment for bone-related diseases. NCT03068351, one clinical trial, found that BET inhibitor RO6870810 may apply for combination therapy for MM. Another clinical trial, NCT02157636, demonstrated that BET inhibitor CPI-0610 can inhibit the progress of MM. But there aren’t clinical trials related to the above SEs-inhibitors initiated. In addition, there aren’t study articles about these two BET-inhibitors, with completed or registered clinical trials carried out, repress the progression of bone-related diseases through SEs reported. So, it’s necessary to verify the clinical treatment effect of the above potential SEs-inhibitors of bone-related diseases through clinical trials.

### CDK Inhibitors Targeting SEs

RNA Pol II, the central enzyme functioning in protein-coding gene transcription, initiation, and elongation, is particularly regulated by CDK7—one of the CDKs (Larochelle et al., 2012). CDK7 can phosphorylate the carboxyl-terminal domain of RPB1 (CTD) of RNA Pol II (RNAPIICTD), thereby regulating transcriptional initiation and pause release, as well as elongation (Palancade and Bensaude, 2003; Glover-Cutter et al., 2009; Larochelle et al., 2012). An increasing number of CDK7 inhibitors have been developed including THZ1 and THZ2. THZ1 is an advanced CDK7 inhibitor that has been reported to have the ability to target the general transcriptional elements and disrupt SEs (Kwiatkowski et al., 2014). Consistent with this, other similar findings of CDK7 inhibitor and SEs have been reported in bone-related cancers. For instance, THZ1 can suppress the proliferation, migration, and invasion of osteosarcoma cells by inhibiting the expression of important SE-containing oncogenic genes (Chen et al., 2018). Sharifnia et al. (2019) found that CDK inhibitors like THZ1 can substantially suppress the growth of chordoma by inhibiting the SEs which is critical for the expression of the important oncogene gene *TBXT* for chordoma. However, Li et al. (2017) reported that the translational significance and clinical application of THZ1 are limited by its short half-life. Compared with THZ1, THZ2 is a newly developed CDK7 inhibitor with a fivefold increase of half-life. Zhang et al. (2020) found that THZ2 can impede the proliferation and metastasis of osteosarcoma via targeting SE-associated oncogenes. Overall, CDK7 inhibition is another promising target treatment therapy for bone-related diseases. Besides CDK7 inhibitors, researchers also suggested that CDK4/6 inhibitors like LEE011 can target SEs. Kennedy et al. (2015) reported that LEE011 can impair the progression of Ewing sarcoma by inhibiting the expression of CDK4/6. In conclusion, SEs are sensitive to CDK inhibition. CDK inhibitors like THZ1, THZ2, and LEE011 also have great therapeutic potential for bone-related diseases.

To date, some clinical trials have been carried out to test the treatment effect of CDK inhibitors on bone-related diseases. For example, NCT01096342, a clinical trial carried out by Kumar et al. (2015) showed that CDK inhibitor-dinaciclib (SCH 727965) can repress the progression of MM. Based on this trial, they reported an article that demonstrates the activity of dinaciclib in relapsed MM (Kumar et al., 2015). But this article didn’t demonstrate that dinaciclib can repress the progression of relapsed MM through influencing SEs in MM. Unfortunately, there aren’t completed or registered clinical trials related to the above SEs-inhibitors initiated. Thus, it’s necessary to verify the clinical treatment effect of the above potential CDK-inhibitors of bone-related diseases through clinical trials.

### Developing New Downstream Target Genes of SEs for Bone-Related Diseases

Super-enhancers inhibitors mainly repress the progression of bone-related diseases by disrupting the SEs associated with bone-related diseases and subsequently influencing the critical oncogenes that are targeted by SEs. There are no studies yet to indicate whether SE inhibitors can be used for the potential treatment of bone-related diseases like osteoporosis, cartilage dysplasia, and RA. In the future, besides carrying out studies using the above two kinds of SEs inhibitors as a treatment approach for bone-related diseases, we should focus more on developing new downstream target genes of SEs for the diagnosis and treatment of bone-related diseases. This approach may ensure sufficient and varied treatment strategies for bone-related diseases.

## Summary and Perspective

The rapid development of bioinformatics technology has driven the study of the genetic mechanism of diseases, which has helped researchers and clinicians to better understand the underlying mechanisms of several disease conditions. Furthermore, some intractable diseases have become less difficult to treat. However, the pathogenic mechanisms of many other complex diseases remain unclear, such as osteosarcoma, Ewing sarcoma, and other bone-related diseases with poor prognosis. Multiple studies have reported novel insights of which many include SEs-associated genes such as *MYC* (Loven et al., 2013; Zhang et al., 2020) LIF, (Lu et al., 2020), and *EWS-FLI1* (Jacques et al., 2016), and SEs-inhibitors with therapeutic effects such as JQ1 (Loven et al., 2013; Lee et al., 2015; Jiang et al., 2017; Chen et al., 2018), THZ1 (Chen et al., 2018; Sharifnia et al., 2019), and THZ2 (Zhang et al., 2020) on diseases like osteosarcoma, Ewing sarcoma, MM, chordoma, and osteoarthritis. However, for osteoporosis, cartilage dysplasia, and RA, only a few therapeutic drugs have been reported that can efficiently target SEs or SEs-associated genes. Besides, there are rarely completed or registered clinical trials that investigate the clinical treatment effect of the potential SEs-inhibitors initiated. Therefore, future research should focus more on the SEs-associated mechanism of the above bone-related diseases and it is so urgent to initiate more clinical trials that testify the potential clinical application of the above-mentioned SEs-inhibitors.

In summary, the above studies on SEs in bone-related diseases focused on the identification of disease-specific SEs and their target genes, followed by verification of the effects of the screened target gene through related *in vitro* and *in vivo* experiments. The specific stepwise method is as follows: first, identifying the bone cancer-related SEs and verifying the most related SEs by techniques such as ChIP-seq. Second, screening target genes associated with the SEs through bioinformatics analysis for building therapies for bone cancer. Finally, testing the potential and accuracy of target genes via *in vitro* and *in vivo* experiments. We believe that the findings from SEs studies will be helpful for the treatment for difficult-to-treat bone-related diseases in the future. However, to a certain degree, as the SEs-related studies most involve in cancer field, the SEs-related studies in skeleton field are rare. So, we can explore the role of SEs in skeleton field in future studies.

The discovery of SEs is a scientific and clinical breakthrough. Many studies have proven that the abnormal expression of SEs is closely related to the disease process. Therefore, research on SEs has gained immense clinical importance. By studying the expression of SEs in diseases, clarifying the pathogenesis of diseases, and finding accurate and efficient target genes, new treatment options can be developed. Thus, it is necessary to study the functions of SEs in bone-related diseases. Recent years have witnessed rapid advancements in technologies like ChIP with high-throughput sequencing (Schmidt et al., 2009) and CRISPR genome editing tool (Burgess, 2013), which offer novel insights into the function and biophysical formation of SEs, as well as the regulatory mechanisms of the targeted gene. With the accumulation of related research, the role of SEs in bone-related diseases is becoming more and more clear. However, as shown above, many studies are on the phenomenon level, and the mechanism researches such as how SEs are regulated and the detailed molecular mechanism of how SEs regulate its target genes are still insufficient. Therefore, the research on the deep and detailed mechanism needs to be strengthened in the future. Besides, the potential of the clinical application of SEs inhibitors in bone-related diseases needs to be further explored. Therefore, future studies of SEs should focus on how to better utilize SEs in the targeted therapy of bone-related diseases in clinical.

## Author Contributions

QL and ZL conceived of this review. ZO and JQ drafted the manuscript. WW and ZO designed the figures. GL and JW designed the tables. JQ revised the manuscript. All authors were involved in the critical revision of the manuscript and approved the final version.

## Conflict of Interest

The authors declare that the research was conducted in the absence of any commercial or financial relationships that could be construed as a potential conflict of interest. The handling editor declared a shared affiliation, though no other collaboration, with the authors at time of review.
